# The impact of digital literacy on individual health: a perspective based on fitness exercise

**DOI:** 10.3389/fpubh.2025.1625235

**Published:** 2025-07-18

**Authors:** Hao Li, Zihan Yang, Jiao Li

**Affiliations:** ^1^School of Business, Xinyang Normal University, Xinyang, China; ^2^Research Institute of the Economic and Social Development in the Dabie Mountains, Xinyang, China

**Keywords:** digital literacy, physical health, exercise behavior, exercise frequency, digital utilization

## Abstract

**Introduction:**

Regular fitness exercise is widely recognized as an effective way to improve residents’ health. In the digital economy era, digital literacy, defined as an individual’s capacity to use internet and digital media technologies for information acquisition, screening, and utilization, played a critical role in shaping fitness participation and health outcomes. This study explored the mechanisms by which digital literacy influenced individuals’ engagement in fitness exercise and their subsequent health status.

**Methods:**

Using longitudinal data from the China Family Panel Studies (CFPS) for 2018, 2020, and 2022, we conducted empirical analysis on the effects and pathways through which digital literacy impacted fitness exercise behavior and health. Our methodology included heterogeneous analysis based on individual characteristics and the structural dimensions of digital literacy.

**Results:**

The findings demonstrated that digital literacy significantly increased individuals’ weekly participation rate and frequency of fitness exercise, although it had no statistically significant effect on the duration of each session. Furthermore, both digital literacy and fitness exercise behavior contributed to improved self-assessed health levels. Heterogeneity analyses revealed that the influence of digital literacy varied across demographic groups, with digital dependence, information acquisition, and digital living environments emerging as key drivers of participation and frequency. The mechanisms linking digital literacy to health improvements included enhanced information access, goal management, social interaction, and online learning.

**Discussion:**

To optimize public health outcomes, policymakers should adopt a people-centered approach, promote the integration of digital technologies with fitness services, and foster environments that support digital literacy development. Addressing disparities in digital literacy within mass fitness initiatives is also crucial for equitable progress.

## 1 Introduction

In today’s digital age, health, as a fundamental human need and the foundation of comprehensive development, holds paramount importance. Regular fitness exercise is a key factor in promoting health, as it not only enhances physical fitness and prevents diseases but also improves mental health and quality of life. Promoting the high-quality development of mass fitness is a crucial cornerstone and key indicator of accelerating the construction of a sports powerhouse. It aligns with the people’s expectation for a healthy body and is an inevitable requirement for achieving a Healthy China. With the rapid development of the digital economy, China places great emphasis on the social application and inclusive services of digital technologies. The “Sports Power Construction Outline,” the “National Fitness Program (2021–2025),” and the “Opinions on Building a Higher-Level Public Service System for National Fitness” all explicitly proposed utilizing digital technologies to enhance the intelligence and scientific level of fitness services, thereby promoting the high-quality development of mass fitness. Centered around 5G technology and integrated with cloud computing, virtual reality, big data, and other emerging digital technologies, these innovations are highly aligned with public fitness services. They optimize people’s service experiences, give rise to new organizational forms and service models, and provide new digital products and services for the fitness domain (1). By 2022, the number of digital fitness users in China had exceeded 200 million, and the online-offline integrated lifestyle had become widely familiar. Fitness activities supported by digital technologies had emerged as a new trend in healthy living (2).

However, while digital technology has served fitness exercises, it has also introduces negative impacts such as the “digital divide,” “digital burden,” and “digital risks” due to variances in individual digital literacy within the public fitness service sphere. This presents new challenges for the application of digital technology in fitness (3). Digital literacy is defined as the comprehensive set of qualities and abilities required for citizens in a digital society to acquire, create, use, evaluate, interact with, share, innovate, and ensure the security of digital information, as well as to adhere to ethical standards in their learning, work, and daily lives (“Enhancing Public Digital Literacy and Skills: Key Points for 2022”). It is an essential component of civic competence in the digital era and a reflection of the basic quality of the nation’s citizens. The level of digital literacy directly impacts the public’s ability to utilize and discern digital technology and information (4). To date, European and American countries had elevated the improvement of citizens’ digital literacy to a national strategic level by establishing relevant policies and plans to cultivate and enhance digital literacy, gradually forming a system driven by both governments and industry organizations (5). In November 2021, China’s Central Cybersecurity and Informatization Commission issued the Outline of Action for Enhancing Public Digital Literacy and Skills, proposing the ambitious goal of “building, by 2035, a society where the digital adaptability, competence, and creativity of all citizens are significantly improved, and where the digital literacy and skills of all citizens reach the level of developed countries.” Against the backdrop of the in-depth implementation of the national fitness and Healthy China strategies, exploring the intrinsic connections between digital literacy and residents’ health exercise and well-being holds paramount practical significance for optimizing public health policies and enhancing the health level of all citizens.

Existing literature has noted the impact of internet use or mobile phone dependence on the participation of different age groups in fitness exercises and on residents’ health. It also emphasizes the differential effects across various age, gender, and household registration groups (6, 7). However, such studies have largely focused on internet or mobile phone use in isolation and have rarely extended to the dimension of digital literacy. Furthermore, existing studies have not combined large-scale micro-level survey data to establish an individual digital literacy evaluation system tailored to the Chinese context. There has also a lack of discussion on the causal relationship between national digital literacy and health through fitness exercise, as well as on heterogeneity analysis and the identification of influencing pathways. In light of this, this paper theoretically reveals the mechanisms through which digital literacy affects individual participation in fitness activities and health. Empirically, we set up econometric models and employed various empirical analysis methods to explore the effects and pathways of digital literacy on individual participation in health exercises and health. This paper differs from previous studies in several ways: First, based on the analysis of the mechanisms through which digital literacy affects public fitness participation, we utilized data from three waves of the China Family Panel Studies (CFPS) in 2018, 2020, and 2022. We extracted indicators reflecting digital literacy from the database, such as individual digital technology usage, and combined this information with indicators of digital economic development in the cities where respondents reside to construct a comprehensive individual digital literacy evaluation system. Second, we built a benchmark regression model to empirically analyze the effects of digital literacy on individual fitness participation and health. We applied robustness tests and instrumental variable methods to verify the accuracy of the empirical results. Third, we examined the heterogeneity of digital literacy’s impact on fitness participation by considering individual characteristics such as gender, age, education level, personal income, health status, and urban location information, as well as the structural components of digital literacy. Fourth, we validated the specific pathways through which digital literacy affects fitness participation, including information acquisition, goal setting and progress management, interaction and community building, and online learning and simulation. Finally, we proposed corresponding policy implications.

The marginal contributions of this paper were as follows:

1. This study has expanded the research perspective to encompass a more comprehensive digital literacy framework. By integrating multi-dimensional factors such as digital device usage, digital application, digital dependence, digital information acquisition, and digital living environment, the research explores their impact on fitness exercise and subsequent health outcomes. This approach addresses the gap in prior studies on the relationship between digital literacy, fitness exercise, and health, offering a richer and more holistic perspective on understanding the drivers of individual fitness behaviors in the digital era.
2. This study has innovatively highlighted the mediating role of fitness exercise in the relationship between digital literacy and residents’ health. It has explicitly identified fitness exercise as a key pathway through which digital literacy influences health. Unlike previous studies that focused solely on the direct effects of digital technology on health or fitness, this study establishes a clear link between digital literacy and health, broadening the theoretical pathways and deepening the academic understanding of their relationship.
3. This study has provided an in-depth analysis of the mechanisms through which digital literacy influences fitness behavior and frequency via multiple specific pathways, including information acquisition, goal setting and progress management, interaction and community building, and online learning and simulation. Compared to previous studies that offered vague overviews of these mechanisms, this analysis is more detailed and thorough. It offers a robust theoretical framework for future research on digital literacy applications in health, thereby advancing the development and refinement of related theories.
4. This study conducts a heterogeneity analysis at two levels: individual characteristics (gender, age, education level, employment status, income level, etc.) and the structural components of digital literacy. It precisely identifies the differential impacts of various groups and elements of digital literacy on fitness exercise. Such a systematic exploration is rare in existing literature. This analysis reveals the complexity and diversity of digital literacy’s influence on fitness exercise, providing a theoretical basis for formulating more targeted health promotion policies and enhancing the precision and effectiveness of policy-making.
5. Utilizing three waves of panel data from the China Family Panel Studies (CFPS), this empirical study differs from previous research relying on foreign data or single cross-sectional data. By using representative domestic data, this study more accurately reflects the actual situation of digital literacy and fitness exercise among Chinese residents. It enriches the empirical evidence on digital literacy and health in developing countries and provides strong data support for domestic policy-making and practice.

## 2 Theoretical analysis

The concept of literacy is developmental, dynamic, and open, and digital literacy continues to deepen and expand with the evolution of digital technologies and the enhancement of public cognition (4). Alkali and Amichai-Hamburger (8) was among the first to interpret digital literacy as the “ability to read and write digital information resources”. With the emergence of Web 2.0 tools and changes in the digital information environment, Martin and Grudziecki (9) posited that digital literacy encompasses the skills required for individuals to engage in production, life, and learning activities within a digital environment. Aviram and Eshet-Alkalai (10) defined digital literacy as the awareness, attitude, and ability of individuals to correctly use digital tools and devices, utilize digital resources, construct new knowledge, innovate media expression, and communicate with others in specific life contexts. They further divided national digital literacy into three developmental stages: the skill stage of correctly using digital tools and devices, the capability stage of acquiring digital resources through these tools, and the stage of creating new knowledge and resources using digital tools. Digital literacy not only includes the ability of individuals to use the internet and digital media technologies to search for, acquire, read, understand, analyze, evaluate, communicate, utilize, and share information resources but also encompasses the ability of individuals to engage in self-criticism, creative thinking, online communication, digital information analysis, and knowledge reorganization within a digital environment (5, 11).

**Figure 1:**
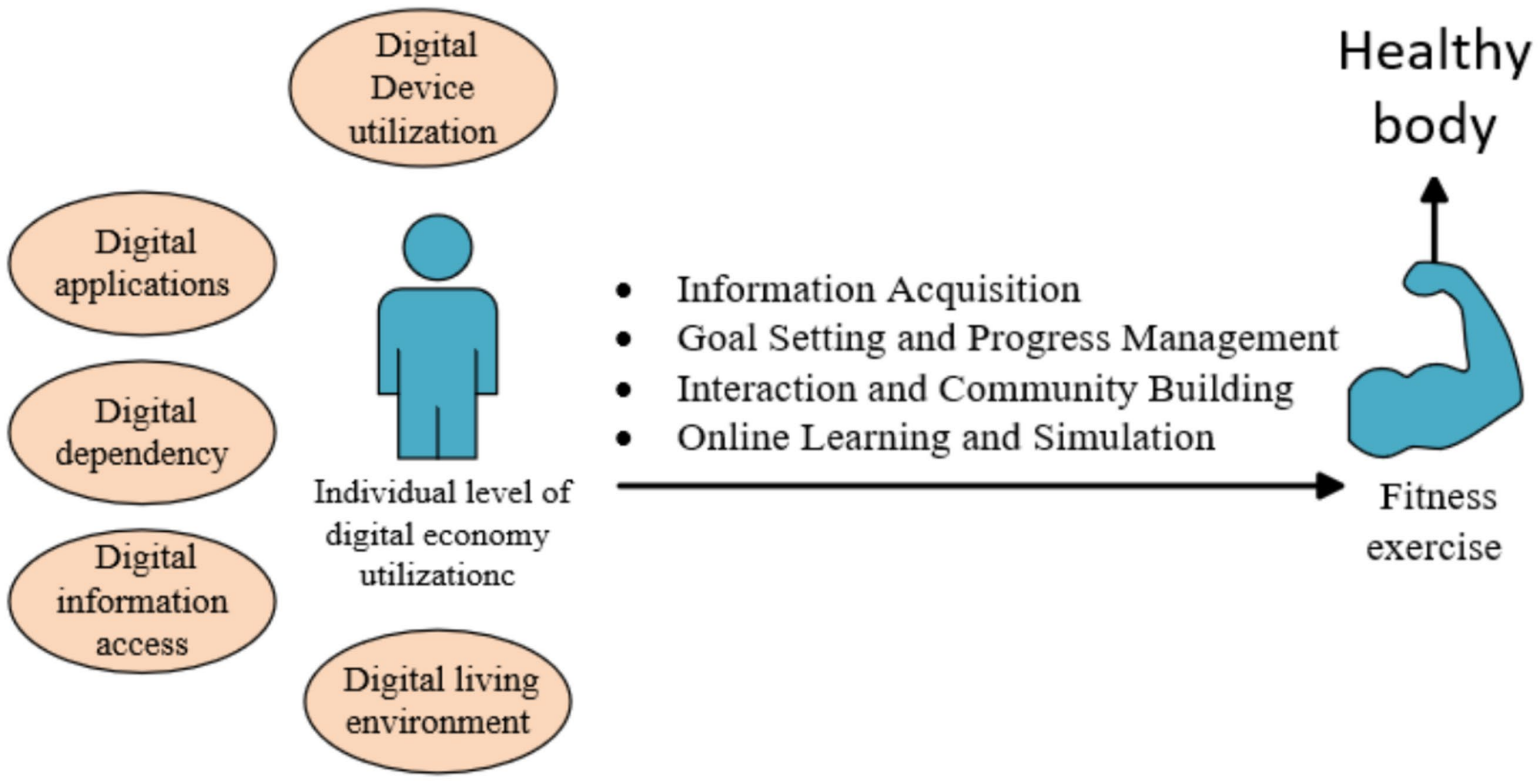
The relationship between digital literacy, fitness exercise, and the health of individual.

The promotional role of digital literacy in fitness exercise is primarily reflected in enhancing information acquisition, goal setting and progress management, social interaction, online learning, and education (4, 7, 12). The relationship between digital literacy, fitness and personal health is shown in Figure 1.

### 2.1 Information acquisition

The rapid development of digital technologies has provided the public with convenient access to fitness information, has maintained and has enhanced cognitive engagement in fitness activities, and has optimized fitness exercise methods. These advancements align with the characteristics of future fitness activities, such as digitalization, scalarization, diversification, and fragmentation, helping to form and strengthen a fitness awareness driven by online information dissemination (12). Individuals with higher digital literacy could more effectively utilize networks, social media, and other channels to obtain sports and fitness knowledge and information. For example, the creation of sports event portals, the dissemination of online fitness knowledge, and the provision of remote fitness guidance have stimulated fitness demand, have enhanced public enthusiasm for fitness activities, and have promoted the acquisition of health-related information, ultimately elevating overall health levels (12). Moreover, regional sports management departments could utilize public fitness service platforms to release relevant governmental information and offer residents a wealth of event activities and fitness guidance services, thereby encouraging local residents to participate in fitness exercises (13).

### 2.2 Goal setting and progress management

Focusing on the application of digital technologies and guided by residents’ fitness needs, digital fitness management platforms—supported by harmonious supply–demand relationships and collaborative efforts from multiple stakeholders—have provided convenient technical support for formulating government fitness plans, allocating fitness resources, maintaining fitness big data, conducting statistical analysis and disseminating fitness data, and enabling intelligent decision-making in fitness initiatives (14). These platforms also have offered technical support for online event registration, fitness consultation, venue booking, online ticket purchasing, and online fitness guidance. Digital fitness management platforms have bridged the gap in fitness resource allocation across regions and among different groups. They assist individuals in self-assessment, accurate evaluation, and the development of fitness intervention measures, guiding individuals toward cultivating healthy behaviors and meeting personalized fitness demands. This, in turn, optimizes the efficiency and management level of mass fitness programs (15). Individuals with higher digital literacy can better utilize digital devices and digital information management platforms to facilitate scientific exercise. For instance, through smart bands and smartwatches, they can set and track exercise goals, record exercise data, monitor their progress and achievements, and adjust exercise plans in real-time. These actions enhance the pertinence and effectiveness of their workouts, thereby helping them better achieve their health objectives (12).

### 2.3 Interaction and community building

Digital platforms have offered exercisers opportunities for communication and sharing, enabling groups with similar fitness interests to form communities. These communities organize group workouts and mutual supervision, thereby enhancing exercise motivation (15). With the innovation of mobile internet and new media technologies, and the widespread application of instant communication functions in fitness domains, intelligent fitness, data check-ins, and fitness social interactions have became popular trends in sports and recreation (16). In the mobile new media environment, self-presentation, sharing, and socializing have become primary motivations for participating in sports activities. The ecology of exercise behavior suggests that individuals’ fitness behaviors are shaped by the interplay of individual and environmental factors (6). Digital media expands residents’ modes of sports participation, broadens interpersonal sports interactions, and enriches sports communication content, thereby increasing sports participation levels (17). Psychological research indicates that digital media can influence residents’ awareness, attitudes, and motivations regarding sports participation. Individuals with higher self-efficacy exhibit greater persistence and tolerance in sports activities, making them more likely to actively engage in exercise and promoting the development and maintenance of healthy behaviors (18).

### 2.4 Online learning and simulation

Equipped with digital literacy, individuals can acquire fitness-related knowledge and skills, including context, culture, language, and communication abilities, from digital resources (19). Participants in physical exercise can utilize online resources to learn new sports knowledge, watch instructional videos, and obtain professional guidance. Digital analysis technologies, including big data and artificial intelligence, provide diversified sports public services that match people’s needs, thereby enhancing their enthusiasm for fitness participation (20). Moreover, digital fitness media can eliminate the limitations of physical space for sports and fitness, presenting virtual sports scenarios to users. This has given rise to new service models, such as online follow-along workouts, interactive guidance, online event experiences, and online fitness activities. Accompanied by elements like commentary, background music, subtitles, animations, and emoticons, these models create immersive sports environments, making fitness more engaging and enjoyable. This helps strengthen individuals’ willingness and perseverance in exercising, thereby promoting health (21). With the advancement of digital technologies, diverse pathways—such as online physical condition analysis, sports health monitoring, scientific fitness guidance, digital fitness equipment, intelligent fitness methods, electronic fitness maps, and fitness news and information—provide effective means for information aggregation, dissemination, and learning. They break through the temporal and spatial boundaries of public fitness, offering multi-scenario solutions to foster fitness awareness, enhance fitness literacy, encourage fitness participation, and invigorate fitness activities among individuals (22).

However, digital fitness media based on network technology has also faced certain challenges. For example, verifying the authenticity of information can be difficult. Variations in digital literacy across different groups lead to uneven levels of understanding and acceptance of digital platforms. In particular, digital vulnerable groups, such as the older adult, minors, individuals with disabilities, and low-income populations, often find it difficult to participate in data collection procedures and obtain useful fitness information and services through digital public fitness platforms. To some extent, this affects residents’ utilization of digital fitness resources and their attitudes toward participating in fitness exercises, thereby influencing the improvement of their health levels (7, 23, 24).

## 3 Research design

### 3.1 Model specification

To investigate the impact of digital literacy on individuals’ participation in health exercises and their health status, this paper constructed the following benchmark regression models based on the aforementioned mechanism analysis:


(1) $$\mathrm{Ex}e_{\mathrm{it}}=\alpha_{\mathrm{it}}+\beta_{\mathrm{it}}Dl_{\mathrm{it}}+\gamma_{\mathrm{it}}X_{\mathrm{it}}+\eta_{i}+\lambda_{t}+\varepsilon_{\mathrm{ij}}$$

(2) $$H{\mathrm{el}}_{\mathrm{it}}=\alpha_{\mathrm{it}}+\beta_{\mathrm{it}}Dl_{\mathrm{it}}+\chi_{\mathrm{it}}\mathrm{Ex}e_{\mathrm{it}}+\gamma_{\mathrm{it}}X_{\mathrm{it}}+\eta_{i}+\lambda_{t}+\varepsilon_{\mathrm{ij}}$$

The dependent variables in our analysis are $\mathrm{Ex}e_{\mathrm{ij}}$ and $H{\mathrm{el}}_{\mathrm{it}}$ , which represent fitness exercise participation and health status, respectively, for individual i at time t . The core explanatory variable, Dl , signifies digital literacy. X encompasses a set of control variables that account for the respondents’ individual characteristics. $\eta_{i}$ captures the individual fixed effects, reflecting the time-invariant heterogeneous attributes of the interviewed individuals. $\lambda_{t}$ represents the time fixed effects, which encapsulate the collective external factor changes experienced by all individuals at the same time. The fixed-effects model allows us to control for time-invariant individual characteristics that might correlate with both digital literacy and fitness behaviors, such as innate health consciousness or personal interest in technology. Additionally, by incorporating time fixed effects, we accounted for common temporal variations that could affect all individuals, such as policy changes or seasonal variations in fitness activities. ε denotes the random disturbance term. Equation 1 primarily assesses how digital literacy influences fitness exercise. Equation 2 evaluates the combined impact of digital literacy and fitness exercise on the health status of the surveyed individuals.

In the analysis of the pathways through which digital literacy affects fitness exercise, this paper employed the following chain-mediated effect model for verification:


(3) $$M_{\mathrm{ijl}}=\alpha_{\mathrm{ij}}+\beta_{\mathrm{ij}}Dl_{\mathrm{ij}}+\gamma_{\mathrm{ij}}X_{\mathrm{ij}}+\eta_{i}+\lambda_{t}+\varepsilon_{\mathrm{ij}}$$

(4) $$\mathrm{Ex}e_{\mathrm{it}}=\alpha_{\mathrm{it}}+\beta_{\mathrm{it}}Dl_{\mathrm{it}}+\chi M_{\mathrm{itl}}+\gamma_{\mathrm{it}}X_{\mathrm{it}}+\eta_{i}+\lambda_{t}+\varepsilon_{\mathrm{ij}}$$

(5) $$\mathrm{He}l_{\mathrm{itl}}=\alpha_{\mathrm{it}}+\theta_{\mathrm{it}}\mathrm{Ex}e_{\mathrm{it}}+\beta_{\mathrm{it}}Dl_{\mathrm{it}}+\chi M_{\mathrm{itl}}+\gamma_{\mathrm{it}}X_{\mathrm{it}}+\eta_{i}+\lambda_{t}+\varepsilon_{\mathrm{ij}}$$

Here, $M_{\mathrm{itl}}$ symbolizes the mediating variables, while l indicates different types of mediating variables. Specifically, Equation 3 analyzes the effect of individual digital literacy on the selected mediating variables, such as information access, goal management, social interaction, and online learning. Equation 4 investigates how both individual digital literacy and these mediating variables affect residents’ participation in fitness exercise. Finally, Equation 5 incorporates individual physical exercise, digital literacy, and various mediating variables to examine their collective impact on residents’ individual health status.

### 3.2 Data selection

This study utilized data from the China Family Panel Studies (CFPS) for the years 2018, 2020, and 2022. The CFPS is a nationally representative longitudinal survey. Its recruitment process utilized a multi-stage random sampling approach. Initially, samples were drawn from diverse regions across China to guarantee population representation. Then, households and their constituent individuals were randomly chosen. The sample encompasses individuals with varying ages, occupations, and socioeconomic backgrounds, thus enabling a thorough analysis of the research questions. Using individual respondent codes, we selected samples that participated in all three survey waves and organized them into panel data for analysis. The utilization of panel data has effectively addressed the issue of weak estimation precision that may arise from small sample sizes (25). The CFPS dataset includes indicators of digital literacy, such as internet usage, purposes of internet use, and the degree of internet dependence. It also contains measures of individuals’ weekly fitness exercise participation and self-assessed health status, which are highly relevant to the research theme of this paper. To ensure the accuracy of the empirical analysis, responses where participants refused to answer or indicated “do not know” were treated as missing data. After excluding cases with missing key variables, we obtained 2,120 valid data points per wave, amounting to a total of 6,360 data points.

#### 3.2.1 Dependent variables

The dependent variables in this study are the respondents’ health status and fitness exercise participation. Health status was primarily measured using self-assessed health levels from survey questionnaires, coded as follows: poor = 1, fair = 2, good = 3, very good = 4, and excellent = 5. Higher scores indicate better health status. Fitness exercise participation was assessed using responses to the survey questions “How many times do you engage in fitness exercise per week?” and “How many hours do you spend on sports exercise per week?” Specifically:

1. Samples that did not exercise within a week were coded as 0, indicating no weekly fitness exercise participation; samples that did exercise within a week were coded as 1, indicating weekly fitness exercise participation.
2. Based on the number of times respondents exercised weekly, a variable for weekly fitness exercise frequency was constructed.
3. The time respondents spent on weekly fitness exercise was organized into a variable for weekly exercise duration.

#### 3.2.2 Main explanatory variables

Based on the definition of digital literacy in the “2022 Work Points for Enhancing the Digital Literacy and Skills of All Citizens” and referencing the “Global Digital Literacy Framework” by UNESCO and the EU’s DigComp 2.2 digital literacy framework, this study integrated the actual digital lives of Chinese residents. By selecting questions from the CFPS questionnaire related to digital device usage and digital technology utilization, along with indicators of digital economic development in the respondents’ cities, we constructed a comprehensive evaluation system for individual digital literacy. This system encompasses five primary indicators: “digital device usage,” “digital application,” “digital dependence,” “digital information acquisition,” and “digital living environment,” along with 23 secondary indicators (9, 26, 27). All selected indicators are positive in nature. Applying the entropy method for weighting, we calculated the comprehensive digital literacy score for respondents. The constructed individual digital literacy comprehensive evaluation indicator system was presented in Table 1.

**Table 1:** Comprehensive evaluation indicator system for individual digital literacy.

Primary indicator	Secondary indicator	Representation method
Digital device usage	Whether using mobile devices to access the internet	Yes = 1, No = 0
	Whether using computers to access the internet	Yes = 1, No = 0
Digital application	Whether playing online games	Yes = 1, No = 0
	Whether shopping online	Yes = 1, No = 0
	Whether watching short videos daily	Yes = 1, No = 0
	Whether engaging in online learning	Yes = 1, No = 0
Digital dependence	Importance of the internet for work	Importance level 1–5
	Importance of the internet for leisure and entertainment	Importance level 1–5
	Importance of the internet for maintaining contact with family and friends	Importance level 1–5
	Importance of the internet for learning	Importance level 1–5
Digital information acquisition	Importance of television as an information channel	Importance level 1–5
	Importance of the internet as an information channel	Importance level 1–5
	Importance of radio as an information channel	Importance level 1–5
	Importance of SMS as an information channel	Importance level 1–5
Digital living environment	Internet penetration rate	Proportion of internet users among the resident population
	Telephone penetration rate	Ownership rate of landline and mobile phones
	Scale of the broadcasting, television, film, and audio-visual recording and production industries	Radio, television, and movies. Number of listed companies involved in film and television recording production
	Employment in the digital industry	Average number of employees in the information transmission, software, and IT services sector at year-end
	Digital inclusive finance index	Peking University Digital Inclusive Finance Index
	Digital medical institutions	Number of medical institutions offering online registration and consultation
	Digital e-government level	Number of government websites
	Government new media presence	Number of official government accounts on new media platforms

Some survey questions in the CFPS were slightly adjusted, and the indicators were processed accordingly. Due to space limitations, detailed calculation results were not presented here.

#### 3.2.3 Control variables

In our study, we meticulously selected and incorporated a set of control variables to enhance the precision and validity of our empirical analysis. These variables, which include individual characteristics such as age, gender, education level, income level, marital status, employment status, insurance status, body mass index, and household registration information, were integrated into our experimental design to account for potential confounding factors that might influence the relationship between digital literacy, fitness exercise, and health outcomes.

Age and gender were included to capture the natural variations in health status and fitness exercise patterns across different demographic groups. Education level and income level were considered due to their potential impact on an individual’s access to and utilization of digital technologies, as well as their health awareness and fitness resources. Marital status and employment status were incorporated to reflect the social and economic contexts that might affect an individual’s time availability and motivation for fitness activities. Insurance status was included to proxy for an individual’s access to healthcare services, which might influence their health outcomes. Body mass index was controlled for to account for the baseline health condition of the respondents, and household registration information was used to capture the urban–rural disparities in fitness infrastructure and digital technology penetration. By including these control variables in our fixed-effects model, we aimed to isolate the main effects of digital literacy on fitness exercise and health status.

In the heterogeneity analysis, we further examined how the impact of digital literacy on fitness exercise varies across different subgroups defined by these control variables. This approach not only strengthens the robustness of our findings but also provides deeper insights into the differential effects of digital literacy on fitness exercise among diverse population segments. Descriptive statistics for the main variables were presented in Table 2.

**Table 2:** Descriptive statistics of main variables.

Variable name	Representation method	Mean	Variance
Exercise behavior	Whether engaged in fitness exercise within a week	0.4912	0.5121
Exercise frequency	Number of times exercising weekly	1.9879	0.7484
Exercise duration	Weekly exercise time (in hours)	2.3127	0.8915
Health status	Poor = 1, Fair = 2, Good = 3, Very Good = 4, Excellent = 5	3.9848	1.9513
Digital literacy	Comprehensive score of digital device usage, digital application, digital dependence, digital information acquisition, and digital living environment	12.3547	3.5871
Age	Respondent’s actual age at the time of survey	41.1985	15.4735
Gender	Male = 0, Female = 1	0.4587	0.4153
Marital status	Unmarried = 0, Married = 1	0.7154	0.4342
Education level	Respondent’s actual years of education	10.2598	4.1009
Employment status	Unemployed = 0, Employed = 1	0.6982	0.5491
Income level	Personal total income (in ten thousand yuan)	5.7284	4.1958
Insurance status	No = 0, Yes = 1	0.7056	0.5162
Body mass index	Weight/Height^2^	22.8096	3.2079
Household Registration	Agricultural household = 0, Non-agricultural household = 1	0.6191	0.5436

## 4 Empirical results and analysis

### 4.1 Benchmark regression results

Based on the established benchmark regression models (1) and the numerical form of the dependent variables, this study first employed logit regression to analyze respondents’ weekly participation in fitness exercise. Subsequently, panel data fixed-effects models were utilized to analyze weekly exercise frequency and duration. The regression results were presented in Table 3.

**Table 3:** The effect of digital literacy on respondents’ participation in fitness exercise.

Variable	Exercise Behavior	Health Status	Exercise Frequency	Health Status	Exercise Duration	Health Status
	(1)	(2)	(3)	(4)	(5)	(6)
Digital literacy	0.0354^***^	0.0041^***^	0.0313^***^	0.0051^***^	0.0212	0.0044^***^
	(4.47)	(5.91)	(5.17)	(6.11)	(1.38)	(5.91)
Fitness exercise		0.3421^***^		0.1241^***^		0.051
		(4.69)		(7.08)		(0.69)
Education Level	0.2452^***^	0.051	0.2135^***^	0.052	0.01053^*^	0.052
	(9.78)	(1.25)	(7.21)	(1.01)	(1.79)	(1.02)
Age	0.7151^***^	0.1224	0.7551^***^	0.1051	0.8082^**^	0.114
	(6.51)	(0.57)	(5.21)	(0.58)	(2.32)	(0.45)
Gender	−0.0425	0.0521^**^	−0.0412	0.0511^**^	−0.0231	0.0451^**^
	(−0.15)	(2.06)	(−1.35)	(2.10)	(−0.24)	(2.36)
Household registration	0.5112^***^	0.651	0.5161^***^	0.605	−0.0215	0.451
	(8.42)	(0.98)	(6.42)	(0.99)	(−0.14)	(1.09)
Marital status	−0.4332^***^	−0.0354^*^	−0.3615^***^	−0.0351^**^	−0.4461^***^	−0.0334^**^
	(−6.38)	(−1.59)	(−4.56)	(−2.01)	(−3.18)	(−1.99)
Employment status	0.0449^**^	−0.1547^***^	0.0511^***^	−0.1427^***^	−0.0171^*^	−0.1471^***^
	(2.09)	(−3.78)	(5.12)	(−4.12)	(−1.76)	(−3.58)
Income level	0.1232	−0.0577^***^	−0.0927^***^	−0.0541^***^	0.0011^**^	−0.06105^***^
	(1.57)	(−4.98)	(−4.51)	(−5.14)	(2.04)	(−4.45)
Insurance status	0.0552^***^	0.1557	−0.0358	0.1532	0.0223	0.1415
	(4.25)	(1.25)	(−1.38)	(1.23)	(0.31)	(1.31)
Body mass index	−0.0121^**^	−0.0057^**^	0.0194	−0.0061^**^	0.0133	−0.0056^*^
	(−2.15)	(−2.41)	(1.21)	(−2.38)	(0.12)	(−1.68)
Constant	−1.051^***^	0.2142	0.0124^***^	0.2112	0.0571^***^	0.2204
	(−4.23)	(0.87)	(10.12)	(0.91)	(8.12)	(1.32)
*R* ^2^	0.0429	0.1241	0.0417	0.1244	0.0271	0.1241
Individual fixed effects	YES	YES	YES	YES	YES	YES
Time fixed effects	YES	YES	YES	YES	YES	YES
*N*	6,360	6,360	6,360	6,360	6,360	6,360

*t*-values were parentheses; *p < 0.1, **p < 0.05, ***p < 0.01.

Column (1) of Table 3 presents the results of the analysis of the impact of digital literacy on respondents’ weekly participation in fitness exercise. The regression coefficients are 0.0354 and significant at the 1% level, indicating that digital literacy can effectively increase respondents’ weekly participation in fitness exercise. In line with incentive theory, the application of digital technology has fostered the integration of sports events and fitness information. Digital software-created fitness knowledge graphs and new media dissemination methods, such as animations, can stimulate and guide the public’s motivation to participate in fitness exercises. This transforms the reluctance to exercise into a desire to engage in physical activity or makes monotonous fitness routines more engaging. Respondents genuinely experience the convenience and benefits brought by digital technology advancements in fitness, thereby increasing their participation in fitness activities (18). Among other control variables, respondents with higher education levels, older ages, urban household registration, unmarried status, formal employment, insurance coverage, and more balanced body status exhibit stronger tendencies to participate in fitness exercises. However, the gender and income level of respondents have no significant impact on their participation in fitness activities.

Column (2) of Table 3 presents the results when considering both digital literacy and fitness exercise behavior on health. The findings indicate that digital literacy and fitness exercise behavior have both effectively promoted individual health levels, consistent with existing research conclusions (28, 29). Following the principle of mediated effect testing, this has further demonstrated that fitness exercise behavior is a crucial transmission pathway through which digital literacy enhances individual health levels. Among the remaining control variables, no significant relationships have been found between education level, age, household registration information, and insurance status with personal health. Additionally, female respondents reported better self-assessed health than male respondents. Unmarried residents had higher self-assessed health levels than married individuals. The unemployed group reported better health than the employed group. Respondents with higher income levels and higher body mass indices perceived their health status as poorer.

Column (3) of Table 3 presents the results of the analysis on the impact of digital literacy on respondents’ weekly fitness exercise frequency. The study finds that digital literacy can effectively increase the frequency of respondents’ weekly fitness exercise. The application of digital technologies and digital applications can remind and guide individuals in their fitness routines. Through online fitness instruction, real-time feedback on exercise outcomes, and goal-setting, these tools effectively stimulate respondents’ enthusiasm and initiative for fitness, enhancing the frequency of their weekly exercise participation (12).

Regression result (4) examines the combined impact of digital literacy and fitness exercise frequency on residents’ self-assessed health. The findings indicate that both digital literacy and fitness exercise frequency have significantly improved residents’ self-assessed health levels. This further confirms that fitness exercise behavior is a key transmission pathway through which digital literacy enhances individual health levels.

Regression result (5) indicates that the total weekly exercise duration of respondents was not influenced by digital literacy. This suggests that while digital literacy can effectively increase the frequency of fitness participation, it does not significantly affect the total time spent exercising weekly. The duration of weekly fitness activities is more likely influenced by factors such as individual work and life schedules, physical condition during each exercise session, and exercise outcomes (30).

Regression result (6) examines the combined impact of digital literacy and exercise duration on respondents’ self-assessed health. The findings show that digital literacy has continued to effectively enhance residents’ self-assessed health levels. However, exercise duration does not significantly affect residents’ self-assessed health. Exercise periods that are too short may fail to achieve the desired fitness outcomes, while exercise exceeding physical capacity can be detrimental to health.

### 4.2 Robustness testing

In the robustness test, to assess the health status of the interviewed residents, this study employed the CFPS survey questionnaire, using “peer-rated health” and “whether you have visited a hospital in the past month” as proxies for self-rated health in the regression analysis. In the previous section, where respondents’ digital literacy was calculated using the entropy method, we re-estimated the comprehensive digital literacy level of respondents using principal component analysis and the horizontal and vertical ranking method to ensure the robustness of the results.

As shown in Table 4, in regression result (1), both residents’ digital literacy and their physical exercise behavior significantly enhanced the peer-rated health status of the interviewed residents. In regression result (2), digital literacy was found to reduce the probability of residents visiting a hospital in the past month at the 10% significance level, while physical exercise behavior has an even more pronounced effect, reducing the probability at the 1% significance level. These findings indirectly suggest that digital literacy and physical exercise behavior can, to some extent, improve residents’ health status. In regression results (3)–(5), regardless of whether the principal component analysis method or the method of stretching the levels both horizontally and vertically was used to recalculate the comprehensive level of individual digital literacy, digital literacy continued to effectively promote the participation and frequency of physical exercise among the interviewed individuals. However, it does not significantly influence the duration of their physical exercise. This further demonstrates the robustness of the study’s findings.

**Table 4:** Regression results of robustness testing.

Digital Literacy	Peer-rated health	Whether you have visited a hospital in the past month	Exercise behavior	Exercise frequency	Exercise duration
	(1)	(2)	(3)	(4)	(5)
Original indicators	0.0647^***^	−0.01^*^			
	(3.24)	(−1.71)			
Exercise behavior	0.478^***^	−0.324^***^			
	(5.14)	(−7.50)			
Principal component analysis			0.0182^***^	0.0208^***^	0.0010
			(4.82)	(5.81)	(0.79)
Horizontal and vertical ranking method			0.1051^***^	0.3041^***^	0.0781
			(3.09)	(5.17)	(1.07)
Control variables	Yes	Yes	Yes	Yes	Yes
*N*	6,360	6,360	6,360	6,360	6,360

*t*-values were in parentheses; *p < 0.1, ***p < 0.01. The table was compiled by the author.

### 4.3 Endogeneity treatment

The previous econometric analysis may have suffered from endogeneity issues due to potential reverse causality. For example, individuals who were enthusiastic about fitness exercise may have been more inclined to use digital auxiliary devices and technologies to guide and record their exercise outcomes. Additionally, respondents’ digital literacy may also have been influenced by other unobservable factors, which could cause endogeneity issues. To reduce the potential impact of endogeneity, this study referred to the research of Sabatini and Sarracino (31) and selected the internet penetration rate at the city level as an instrumental variable for respondents’ digital literacy, employing the 2SLS method for regression analysis. The higher the internet penetration rate, the greater the possibility that individuals would proficiently master digital skills. This satisfied the correlation assumption between digital literacy and the instrumental variable. Furthermore, the internet penetration rate at the city level had no direct relationship with respondents’ fitness exercise participation, satisfying the exogeneity assumption. The regression results using the instrumental variable method were presented in Table 5.

**Table 5:** Regression test results using instrumental variables.

	Digital literacy	Exercise behavior	Exercise frequency	Exercise duration
	(1)	(2)	(3)	(4)
Digital literacy		0.0051^**^	0.0481^***^	−0.0258
		(2.34)	(5.15)	(−0.87)
First-stage regression result	0.0351^***^			
	(14.12)			
Kleibergen-Paap *F*-value	28.13			
DWH test	0.0040			
Control Variables		Yes	Yes	Yes
*N*		6,360	6,360	6,360

*t*-values were in parentheses; **p < 0.05, ***p < 0.01.

The regression results in Table 5 indicated that after controlling for endogeneity using the instrumental variable method, digital literacy remained effective in enhancing respondents’ weekly fitness exercise participation and frequency, while it does not significantly affect weekly exercise duration. These findings were consistent with the prior conclusions.

### 4.4 Heterogeneity analysis

#### 4.4.1 Heterogeneity analysis based on individual characteristics of residents

Given space constraints and prior regression results indicating that digital literacy exhibited a consistently similar impact on respondents’ fitness exercise behavior and weekly exercise frequency, along with its negligible effect on weekly exercise duration, this study primarily examined the heterogeneous effects of digital literacy on fitness exercise behavior. Specifically, we divided the sample into distinct subgroups based on individual characteristics such as gender, age, education level, income level, employment status, and health status. For each subgroup, we conducted separate regression analyses using the same model specification as in the benchmark regression. This approach allowed us to identify whether the effect of digital literacy on fitness exercise differs significantly across these subgroups. The regression results are presented in Table 6.

**Table 6:** Heterogeneity analysis of individual characteristic differences.

Fitness exercise behavior						
Digital literacy	Male	Female	Rural	Urban	Unemployed	Employed
	(1)	(2)	(3)	(4)	(5)	(6)
	0.0326^***^	0.0123^***^	0.0031	0.0354^***^	0.0308	0.0354^***^
	(5.07)	(4.39)	(1.03)	(7.58)	(0.56)	(4.08)
	Minors	Middle-aged	Older adult	Primary Education	High School	Bachelor’s and Above
	(7)	(8)	(9)	(10)	(11)	(12)
	0.0224	0.0325^***^	0.0069^*^	0.0016	0.0247^***^	0.04286^***^
	(1.63)	(4.31)	(1.81)	(0.24)	(5.12)	(6.43)
	Low-income	Middle-income	High-income	Eastern	Central	Western
	(13)	(14)	(15)	(16)	(17)	(18)
	0.0234^***^	0.03262^***^	0.0192	0.0355^***^	0.0295^***^	0.0242^**^
	(5.12)	(7.23)	(1.46)	(5.23)	(4.25)	(2.48)
	Low-frequency Group	Medium-frequency Group	High-frequency Group	Low Health Level	Medium Health Level	High Health Level
	(19)	(20)	(21)	(22)	(23)	(24)
	−0.0244^***^	0.0103^***^	0.0251^***^	0.0521^***^	0.04235^***^	0.0222^***^
	(−7.24)	(6.51)	(7.21)	(5.13)	(4.32)	(4.52)

*t*-values were in parentheses; *p < 0.1, **p < 0.05, ***p < 0.01. Regression results for other control variables are omitted.

Table 6 presents the results of the heterogeneity analysis of the impact of digital literacy on fitness exercise behavior, considering the diverse characteristics of respondents. Results (1) and (2) indicated that digital literacy does not differ in its impact on fitness exercise behavior between genders; it effectively enhances the fitness participation of both males and females. Regression results (3) and (4) showed that digital literacy does not significantly affect the fitness exercise behavior of rural household registrants but significantly boosts the fitness participation of urban household registrants at the 1% significance level. Urban areas typically have more developed digital and fitness infrastructure, higher levels of public fitness service provision, and a stronger sense of belonging among urban residents, all of which contribute to increased fitness participation (18). Regression results (5) and (6) suggested that formally employed individuals are more inclined to engage in fitness activities. In regression results (7)–(9), digital literacy does not significantly affect the fitness exercise behavior of minor residents. It significantly enhances fitness participation among middle-aged groups at the 1% level and among older adult groups at the 10% level. Minors, being in a critical learning phase with limited exposure to digital devices, showed no significant impact of digital literacy on their fitness behavior. Meanwhile, older adult individuals, driven by health considerations and ample leisure time, may already have established exercise habits. The “digital divide” also weakens the influence of digital literacy on their fitness participation (7). Regression results (10)–(12) indicated that digital literacy is more effective in promoting fitness exercise among individuals with high school education or above. Those with higher education levels are more aware of the health benefits of exercise and generally possess higher digital literacy, making them more responsive to its influence. Regression results (13)–(15) showed that digital literacy is more effective in promoting fitness among low- and middle-income groups. This may have been because higher-income individuals can access more specialized fitness services and may not have relied on digital devices for exercise guidance. Regression results (16)–(18) suggested that digital literacy does not significantly affect fitness participation across different regions. The universality, convenience, and popularization of digital devices and technologies mean they do not vary significantly by geographical location (24). Regression results (19)–(21) revealed a significant negative correlation between digital literacy and fitness exercise behavior among low-frequency exercisers at the 1% level, while a significant positive correlation exists among medium- and high-frequency exercisers. This implied that the entertainment attributes of digital technologies may have further reduced the motivation of inactive individuals to exercise, decreasing their participation in fitness activities. However, for those already engaged in regular exercise, digital literacy can further enhance their fitness participation (16). Regression results (22)–(24) indicated that digital literacy effectively promotes fitness exercise behavior across all health levels.

#### 4.4.2 Heterogeneous analysis of digital literacy component structure

Digital literacy, a critical skill in the digital age for residents to use digital technologies, may have varying effects on individual fitness exercise due to its different components. To clarify which specific aspects of digital literacy affect fitness participation, this study examined how different aspects of digital literacy—such as digital device usage, digital application, digital dependence, digital information acquisition, and digital living environment—affect individuals’ fitness exercise behavior. The regression results are presented in Table 7.

**Table 7:** Heterogeneous analysis of digital literacy component structure.

	Exercise behavior	Exercise frequency	Exercise duration
	(1)	(2)	(3)
Digital device usage	0.0351	−0.0424^***^	−0.0514
	(1.23)	(−3.74)	(−1.32)
Digital application	−0.0242^***^	−0.0523^***^	−0.0323^**^
	(−5.06)	(−4.36)	(−2.37)
Digital dependence	0.0569^*^	0.0521^**^	0.0356
	(1.72)	(2.05)	(0.52)
Digital information acquisition	0.0367^***^	0.0353^***^	0.0230^*^
	(5.19)	(4.69)	(1.90)
Digital living environment	0.0795^***^	0.0547^***^	0.0341
	(6.32)	(4.12)	(0.81)

*t*-values were in parentheses; *p < 0.1, **p < 0.05, ***p < 0.01. Regression results for other control variables are omitted.

Table 7 column (1) presents the results of the heterogeneous analysis of the effects of digital literacy components on individuals’ fitness exercise behavior. It was seen that digital dependence, digital information acquisition, and digital living environment significantly enhance individuals’ weekly fitness exercise behavior. The influence of digital device usage on individuals’ weekly fitness exercise behavior was not significant. Digital application, on the other hand, reduces individuals’ weekly fitness exercise behavior. Digital literacy components, such as digital technology application, digital sports information dissemination, and digital fitness scenarios, effectively promote individuals’ participation in fitness activities. However, individuals’ use of digital apps can easily lead to addiction to entertainment and leisure activities facilitated by digital technologies, such as online games and short videos, thereby reducing their weekly fitness exercise behavior (6).

Table 7 column (2) presents the results of the analysis of the impact of digital literacy components on individuals’ weekly fitness exercise frequency. The findings indicated that digital dependence, digital information acquisition, and digital living environment significantly increase individuals’ weekly fitness exercise frequency, while digital device usage and digital application reduce it.

Table 7 column (3) presents the regression results of the impact of digital literacy components on individuals’ weekly fitness exercise duration. Except for digital information acquisition, which marginally increases weekly fitness exercise duration at the 10% significance level, digital application marginally reduces it. Digital device usage, digital dependence, and digital living environment do not significantly affect weekly fitness exercise duration. Digital information acquisition effectively reminds individuals of the importance of health exercise, thereby moderately increasing the time individuals allocate to fitness activities.

### 4.5 Pathway testing

Previous theoretical analysis indicates that digital literacy can influence individual fitness exercise through mechanisms such as information access, goal management, social interaction, and online learning, thereby enhancing individual health levels. Based on this, this study employed a chain-mediated effect model to examine the pathways through which digital literacy affects fitness exercise and ultimately health levels. Information access was proxied by “leisure internet usage time,” as more leisure internet time correlates with greater exposure to internet information and richer access to fitness knowledge. Goal management was measured by responses to the survey question “Do you plan your schedule?” Individuals who plan their daily activities are more likely to adhere to fitness plans. Social interaction was proxied by the question “how sociable are you,” as higher sociability indicates more frequent interpersonal interactions and greater social integration, increasing the likelihood of group fitness participation (16). Online learning and simulation were represented by the variable “the number of times you engage in online learning per week” from the CFPS survey. In the chain-mediated effect model, bootstrapping with 2,000 samples was used to calculate confidence intervals. A chain-mediated effect was considered significant if the confidence interval does not include zero. The regression results are presented in Table 8.

**Table 8:** Pathway analysis of digital literacy’s impact on individual fitness exercise.

Mediating Variable	Exercise Behavior	Lower	Upper	Exercise Frequency	Lower	Upper
Information access	0.0326^***^	0.36197	1.38964	0.0514^***^	0.16925	1.49171
	(4.35)			(5.91)		
Goal Management	0.0051^***^	1.82739	3.59214	0.0041^***^	0.45328	1.97653
	(4.01)			(3.10)		
Social interaction	0.0017^***^	0.38271	1.84526	0.0421^***^	5.21847	7.36482
	(7.65)			(5.09)		
Online Learning	0.0021^**^	1.35411	2.51259	0.0031^***^	3.37879	3.98126
	(2.21)			(3.24)		

*t*-values were in parentheses; **p < 0.05, ***p < 0.01. Regression results for other control variables are omitted. Given the insignificant effect of digital literacy on residents’ fitness exercise duration in previous studies, the pathways for this variable are not examined here.

The left side of Table 8 presents the regression results of how digital literacy affected fitness exercise behavior through respective mediating variables and in turn impacted health. The findings show that digital literacy effectively improved individuals’ information access, goal management, social interaction, and online learning, thereby promoting residents’ fitness exercise behavior and enhancing health levels.

Similarly, the right side of Table 8 presents the regression results of how digital literacy affected fitness exercise frequency through respective mediating variables and subsequently impacted health. The results indicate that digital literacy effectively enhanced individuals’ information access, goal management, social interaction, and online learning, thus increasing residents’ fitness exercise frequency and improving health levels.

## 5 Conclusion and recommendations

### 5.1 Conclusion

The findings of this study on the impact of digital literacy on residents’ fitness exercise behavior and health are as follows. The study utilized three waves of data from the China Family Panel Studies (CFPS) for 2018, 2020, and 2022. The conclusions were as follows:

1. Digital literacy has significantly enhanced residents’ participation in fitness exercise and exercise frequency, as well as self-assessed health levels. However, its impact on exercise duration per session was not significant. This suggested that digital literacy primarily boosts residents’ willingness and regularity in exercising, thereby improving self-assessed health levels. Exercise duration is more influenced by factors such as individual physical condition and time arrangement, and only moderate exercise duration can effectively enhance residents’ health levels.
2. The impact of digital literacy on different resident groups has varied significantly. Urban residents, the employed, middle-aged and young adults, those with higher education, middle- and low-income groups, and active fitness participants have been more motivated to engage in fitness activities due to improved digital literacy. This may have been related to these groups’ acceptance of digital technologies, health needs, and lifestyles.
3. Digital literacy has influenced residents’ fitness exercise and health levels through pathways such as information access, goal management, social interaction, and online learning. It has helped residents access fitness information, scientifically plan exercise goals, encourage fitness behavior through online interactions, and learn fitness knowledge via online resources, thereby increasing the willingness and frequency of exercise.

This study concentrated on the extensive and diverse population of Chinese residents, yielding significant practical insights. Amid China’s accelerated digitalization and profound shifts in residents’ lifestyles, digital literacy has emerged as a crucial determinant of fitness participation and health outcomes. However, the following limitations should be acknowledged. First, the data concluded in 2022 and may not fully capture the rapid progression of digital technologies. Moreover, the data lacked detailed information on specific types and intensity of fitness exercises, and the measurement of digital literacy could be further refined. Second, while instrumental variable methods were employed to address endogeneity issues, causal inferences remain challenging, and model assumptions may not fully align with real-world complexities. Third, the study primarily focused on the general resident population and paid insufficient attention to special groups such as the older adult and children. Health outcomes were measured indirectly, without an in-depth exploration of digital literacy’s comprehensive impact on long-term health indicators. Fourth, the study is limited in that it solely employs a panel data fixed effects model to control for major health events potentially occurring during the study period. Attention will be given to better integrating these factors into the study in future research. Lastly, the study did not analyze micro-geographical differences within the same region or provide international comparisons. These limitations offer directions for future research.

### 5.2 Policy recommendations

This study examined how digital literacy among Chinese residents affected their fitness exercise behavior and subsequent health outcomes, offering vital insights for developing targeted health promotion policies, encouraging comprehensive fitness participation, and boosting public health levels.

(1) The government should integrate digital literacy education into public health strategies. It needs to promote digital knowledge and skills through diverse online and offline channels. This will enhance residents’ digital literacy and their capacity to access health information and engage in fitness activities. At the same time, the government should regulate digital health platforms to ensure the accuracy of health information and create a positive digital environment for residents.

(2) Communities should organize diverse digital fitness activities based on their unique characteristics and residents’ needs. They can establish convenient fitness facility networks to encourage participation. Additionally, building digital fitness communities can promote communication and mutual supervision among residents, thereby enhancing their enthusiasm for fitness.

(3) At the societal level, it is essential to encourage technology companies to develop digital fitness products and services tailored to different groups to meet diverse fitness needs. The media should strengthen promotion efforts to raise residents’ awareness and acceptance of digital fitness, create a positive atmosphere around it, and guide residents toward adopting healthy lifestyles.

## Data Availability

The raw data supporting the conclusions of this article will be made available by the authors, without undue reservation.
